# Our New York Letter

**Published:** 1888-10

**Authors:** 


### OUR NEW YORK LETTER.

A NEW FAD IN NEW YORK—THE MAUSOLEUM SAFE DEPOSIT COMPANY—DEATH OF DR. THOMAS T. SABINE—A NEW HONOR CONFERRED ON DR. FORDYCE BARKER—THE SUMMER VACATION AT AN END.

*To the Editors of the Atlanta Medical and Surgicat Journal:*

Mr. John G. Myers, of this city, has lately suggested a new plan for disposing of the dead, by which the bodies are to be subjected to a "dry process" and desiccated. It is proposed to have a large building, constructed like a safe deposit, with corridors, into which will open numerous vaults, and the undertaking is now spoken of as the "Mausoleum Safe Deposit Plan." It is intended to be inexpensive and to obviate the possibility of bodies being stolen. Besides this, it is claimed, from a sanitary point of view, that the dead remains cannot become in any way a source of danger to the living, and also that the indications of crime will not be destroyed, as would occur by cremation. It is intended to construct an attractive building, fire-proof and very substantial, which may be several stories high, and able to contain from one to ten thousand vaults. It is intended to desiccate the bodies somewhat in the following manner: The vaults being hermetically sealed, a current of dry air is conducted into them and through the casket, passing finally by a forced draft to a furnace in the building, where all gases and vapors taken up by the air current are burned. Thus there will be no effusion of odors or noxious principles. It is said that such a process as this will in a short time naturally preserve the remains. Experiments in this direction were recently made at the University Medical College, which seem to show satisfactorily that the invention is a valuable one. A metallic casket was used containing a body which at death weighed 175 pounds. After two weeks the bulk had apparently disappeared to the extent of one-quarter. A pipe at one end of the casket admitted the cold, dry air, which passed all around the body and out through another opening leading to the furnace. The draught is furnished by the heat from the furnace. There was also a small opening under the casket for all liquids to run off, these also being conveyed to the furnace. A number of New Yorkers are interested in the scheme, and there is already talk of building an extensive mausoleum here.

Since my last communication, Dr. Thos. T. Sabine has passed over to the great majority. He will doubtless be remembered by many of your readers as professor, for a number of years, of that very dry subject to most of us, anatomy, at the College of Physicians and Surgeons—a man who understood and taught his branch well, and generally managed to ask some rather perplexing questions at examination time. Dr. Sabine ranked high as a surgeon and an anatomist, but for a great number of years he has been in delicate health, and for a long time his ability to attend a large hospital practice and occupy his chair at the college with so much credit has been a marvel to all who knew him.

The degree of LL. D. has again been conferred upon Dr. Fordyce Barker, of this city. Columbia College was the first institution to honor him in this way, the University of Edinburgh following. He has now received this degree for the third time, as the Glasgow University has recently tried to still further distinguish him.

At the time of my writing the summer vacation is about over, the doctors who have spent a period out of town are beginning to return, and the medical societies will for the most part soon begin their fall work.

Items of medical news are scarce at this time, but in a few weeks all the wheels will be revolving again and I hope to be able to give you a more interesting communication next month.

*New York, Sept. 8, 1888.* A. F.

